# Utility of APOE testing for reducing ARIA under probabilistic stopping rates to treat with anti-amyloid therapy for ε4-homozygote patients: A simulation study

**DOI:** 10.1016/j.jarlif.2026.100059

**Published:** 2026-01-24

**Authors:** Kenichiro Sato, Yoshiki Niimi, Masanori Kurihara, Ryoko Ihara, Atsushi Iwata, Takeshi Iwatsubo

**Affiliations:** a Dementia Inclusion and Therapeutics, The University of Tokyo Hospital, Hongo 7-3-1, Bunkyo-ku, Tokyo, 113-8655, Japan; b Unit for Early and Exploratory Clinical Development, The University of Tokyo Hospital, Hongo 7-3-1, Bunkyo-ku, Tokyo, 113-8655, Japan; c Department of Neurology, Tokyo Metropolitan Institute for Geriatrics and Gerontology, Sakaecho 35-2, Itabashi-ku, Tokyo, 173-0015, Japan; d National Center of Neurology and Psychiatry, Ogawahigashi-cho 4-1-1, Kodaira-shi, Tokyo, 187-8551, Japan

**Keywords:** APOE, Anti-amyloid treatment, ARIA, Number needed to test, Alzheimer's Disease, Shared decision-making

## Abstract

**Background:**

*APOE* ε4/ε4 genotype increases the risk of Amyloid-Related Imaging Abnormalities (ARIA) from anti-amyloid antibody treatment (AAT). While guidelines recommend testing, its practical utility depends on the resulting probability (*p*) that treatment is actually withheld for ε4-homozygotes, which varies significantly across clinical settings.

**Objectives:**

To quantify the Number Needed to Test (NNT) to prevent one ARIA event as a function of *p* of withholding AAT in ε4/ε4 patients.

**Design:**

A Bayesian simulation study using a Beta-Binomial model to analyze genotype-stratified contingency tables.

**Setting:**

Data were derived from two published, phase 3 clinical trials: Clarity-AD (lecanemab) and TRAILBLAZER-ALZ 2 (donanemab).

**Participants:**

Aggregate data from source trials.

**Intervention:**

Simulation of varying treatment discontinuation probability *p* from 0 (none) to 1 (universal for ε4-homozygotes).

**Measurements:**

NNT to prevent one ARIA event (any ARIA-E, any ARIA-H, and symptomatic ARIA-E) and the fractional reduction in total ARIA events as a function of *p*.

**Results:**

NNTs increased (worsened) significantly as *p* decreased. Under the most conservative policy (*p* = 1), the median NNT to prevent one any ARIA-E event was 20–30 (lecanemab) and 15–25 (donanemab), yet this only reduced total ARIA events by 10–30%. The NNT to prevent one symptomatic ARIA-E (lecanemab) was substantially higher, at 70–90 (at *p* = 1).

**Conclusions:**

The direct safety impact of *APOE* testing for ARIA mitigation is limited, even under universal discontinuation policies. Its primary value lies in supporting shared decision-making and operational planning rather than as a standalone safety lever.

## 1 Introduction

Over the past several years, anti-amyloid antibody treatment (AAT) [1], including lecanemab [2] and donanemab [3], have moved from a contested regulatory inflection point toward broader clinical implementation. The accelerated approval of aducanumab [4] based on an amyloid surrogate intensified debate about effect size, clinical meaningfulness, and the real-world feasibility of treatment programs in the presence of treatment-related adverse events, most notably amyloid-related imaging abnormalities (ARIA) [5].

In this context, ARIA has functioned not only as a safety outcome but also as a practical constraint that shapes whether and how anti-amyloid therapy can be delivered in routine care. Subsequent phase 3 trials for lecanemab (Clarity-AD) [2] and donanemab (TRAILBLAZER-ALZ 2) [3] provided more conventionally positive efficacy evidence, yet both agents retain substantial ARIA risk and require MRI monitoring and risk counseling [6,7]. As a result, health systems have adopted heterogeneous approaches to implementation, including differences in how patient-level risk information is incorporated into treatment decisions. At this juncture, it is timely to quantify what risk-informed withholding can realistically accomplish in practice.

*APOE* ε4/ε4 status is a well-established risk factor for ARIA during AAT [5]. Current Appropriate Use Recommendations (AUR) and package insert by FDA thus recommend *APOE* genotyping prior to treatment initiation [6,7]. Depending on the test result, there would be cases where, after discussion with the patient, AAT is pursued or not pursued; in some facilities, AAT may not be provided as part of safety management policy [8]. In some jurisdictions (e.g., EU/UK), lecanemab and donanemab are not approved for ε4-homozygote patients [9,10], which means that testing must be performed before treatment, and if ε4/ε4 is identified, treatment will invariably not be given.

In this point of view, in settings like the EU/UK where ε4-homozygotes are deterministically not treated, the ARIA-risk reduction attribuTable to testing occurs by the margin contributed by excluding ε4/ε4 cases (i.e., relative to those who are not ε4 homozygotes). Conversely, if in practice many ε4 homozygotes end up receiving treatment (e.g., only patients with high treatment motivation may remain at the pre-treatment stage, the way of confirmed consent provided, and institutional policy), *APOE* testing will not have effective influence on the treatment policy; consequently, testing itself yields little contribution in ARIA-risk reduction.

Thus, the extent to which *APOE* testing can reduce ARIA risk depends on how often treatment is actually withheld for ε4/ε4 patients across settings. This variability makes the practical value of *APOE* testing a specific decision problem for clinician-patient counseling, health-system planning for MRI/infusion workflows, and policy/payer implementation requirements. Here we aim to calculate a quantitative indicator, the number needed to test (NNT) [11] to prevent one ARIA event, and the reduction fraction relative to predicted ARIA incidence; we then formalize how these relate to treatment practice for ε4 homozygotes, conduct simulations, and qualitatively interpret the findings.

## 2 Methods

### 2.1 Data and outcomes

Genotype-stratified contingency tables (ARIA vs no ARIA by treatment vs placebo, within ε4/ε4 and non-ε4/ε4 strata), as illustrated in Table 1, were abstracted from Clarity-AD (lecanemab) [2] and TRAILBLAZER-ALZ 2 (donanemab) [3]. Outcomes considered included any ARIA-E, any ARIA-H, and symptomatic ARIA-E (symptomatic ARIA-E available for lecanemab; symptomatic ARIA-H by genotype was unavailable).

**Table 1:** Trial Contingency Table.

*APOE* Genotype	Arm	w/ ARIA	w/o ARIA	Subtotal
ε4/ε4	Active	*Ah*	*Bh*	*Ah+Bh*
ε4/ε4	Placebo	*Ch*	*Dh*	*Ch+Dh*
NOT ε4/ε4	Active	*An*	*Bn*	*An+Bn*
NOT ε4/ε4	Placebo	*Cn*	*Dn*	*Cn+Dn*

Abbreviations: ARIA, Amyloid-Related Imaging Abnormalities.

### 2.2 Formulation of NNT

Scope of analysis (risk-focused): This study quantifies the direct safety and operational impact of *APOE*-guided withholding through ARIA outcomes and MRI burden only. We do not model clinical benefit (e.g., cognitive/functional outcomes), quality-of-life, costs, or cost-effectiveness, and we make no assumptions about differential efficacy by *APOE* genotype. The withholding probability *p* is treated as an operational parameter that emerges from real-world practice and counseling rather than a normative “optimal” policy. Accordingly, the present simulations are not a full decision-analytic model; they provide a risk-focused quantitative component that can be combined with separate evidence on benefit if desired.

Let π be the ε4-homozygote prevalence in the treated population, and p the *post-hoc* site-level probability of withholding therapy when ε4/ε4 is identified. We define drug-attribuTable risk differences within ε4/ε4 and non-ε4/ε4 strata as RD_h_ and RD_n_, respectively. These ARIA risks are obtained from trial contingency Table (Table 1), as follows:

Table 2

• ε4 homozygotes, Active arm: r_hA_ = A_h_ / (A_h_ + B_h_)
• ε4 homozygotes, Placebo arm: r_hP_ = C_h_ / (C_h_ + D_h_)
• non-ε4 homozygotes, Active arm: r_nA_ = A_n_ / (A_n_ + B_n_)
• non-ε4 homozygotes, Placebo arm: r_nP_ = C_n_ / (C_n_ + D_n_)

**Table 2:** Contingency Tables from Previous Trial Results.

Study	genotype	arm	w/ any ARIA-E	w/o any ARIA-E	w/ any ARIA-H	w/o any ARIA-H	w/ symptomatic ARIA-E	w/o symptomatic ARIA-E
Lecanemab (Clarity-AD) [2]	ε4/ε4	Active	*46*	*95*	*55*	*86*	*13*	*128*
	ε4/ε4	Placebo	*5*	*128*	*28*	*105*	*0*	*133*
	NOT ε4/ε4	Active	*67*	*690*	*100*	*657*	*12*	*745*
	NOT ε4/ε4	Placebo	*10*	*754*	*53*	*711*	*0*	*764*
Study	genotype	arm	w/ any ARIA-E	w/o any ARIA-E	w/ any ARIA-H	w/o any ARIA-H		
Donanemab (TRAILBLAZER-ALZ2) [3]	ε4/ε4	Active	*58*	*85*	*72*	*71*		
	ε4/ε4	Placebo	*5*	*141*	*30*	*116*		
	NOT ε4/ε4	Active	*143*	*564*	*194*	*513*		
	NOT ε4/ε4	Placebo	*11*	*713*	*85*	*639*		

Abbreviations: ARIA, Amyloid-Related Imaging Abnormalities.

Then, drug-attribuTable risk differences (RD) are:

• RDh = rhA - rhP
• RDn = rnA - rnP

In the above formulation, π = (A_h_ + B_h_ + C_h_ + D_h_) / N_total_, and p is an operational parameter defined as the posterior probability of withholding treatment if ε4/ε4 is identified (e.g., in the EU/UK, *p* = 1; if results are not considered at all, *p* = 0). In practice, p is obtained *a posteriori* from accumulated treatment experience. If *APOE* testing is performed and, based on the result, treatment is withheld, we define the absolute risk reduction (ARR) per one test as the degree to which adverse events (i.e., ARIA, additional MRI procedures) are avoided per test. Then the NNT to prevent one adverse event is NNT = 1 / ARR.

Now we define NNT: reduction in total ARIA events under a policy that withholds treatment in ε4/ε4 with probability p. Because ARR ∝ p·π, NNT scales inversely with π and improves linearly with p, allowing site-specific recalibration if local ε4/ε4 prevalence differs from trials.

For derivation of NNT, baseline (no APOE testing; treatment given irrespective of genotype) risk is risk_base_ = π·rhA + (1 - π)·rnA. Under policy (APOE testing performed; if ε4/ε4, treatment is withheld with probability p), risk is risk_policy_ = π·{(1 - p)·rhA + *p*·r_hP_} + (1 - π)·rnA. Thus, the absolute risk reduction is ARR = risk_base_ - risk_policy_ = *p*·π·(r_hA_ - r_hP_) = *p*·π·RD_h_, yielding NNT = 1 / (p·π·RD_h_). The fractional reduction in total ARIA events is Reduction = ARR / risk_base_ = {p·π·RD_h_} / {π·r_hA_ + (1 - π)·r_nA_} ∝ p.

### 2.3 Simulation procedure

Our simulation was made based on the following procedure: (i) Parameters A-D (trial cell counts) are referenced from Clarity-AD (lecanemab) [2] and TRAILBLAZER-ALZ 2 (donanemab) [3]. (ii) In each cell for the contingency tables, we conducted Monte-Carlo simulation: apply a Jeffreys prior Beta (0.5, 0.5) and use a Beta-Binomial model to obtain r_hA_, r_hP_, r_nA_, r_nP_ (e.g., *r_hA_* ∼ *Beta(A_h_+0.5, B_h_+0.5)*). (iii) Perform repeated sampling to generate RDh, RDn, π sequentially. (iv) From these, compute ARR, NNT, and reduction proportions, and report point estimates and 95 % credible intervals (CrIs). If each ARIA event triggers on average k additional brain MRI scans, we can also define NNT_MRI_ = NNT/k to express the testing effort required to avert one MRI.

### 2.4 Ethics

This study was approved by the University of Tokyo Graduate School of Medicine Institutional Ethics Committee (ID: 2025264NI). No informed consent was required as it uses publicly distributed data only.

## 3 Results

### 3.1 NNT for avoiding any ARIA

**Fig. 1:**
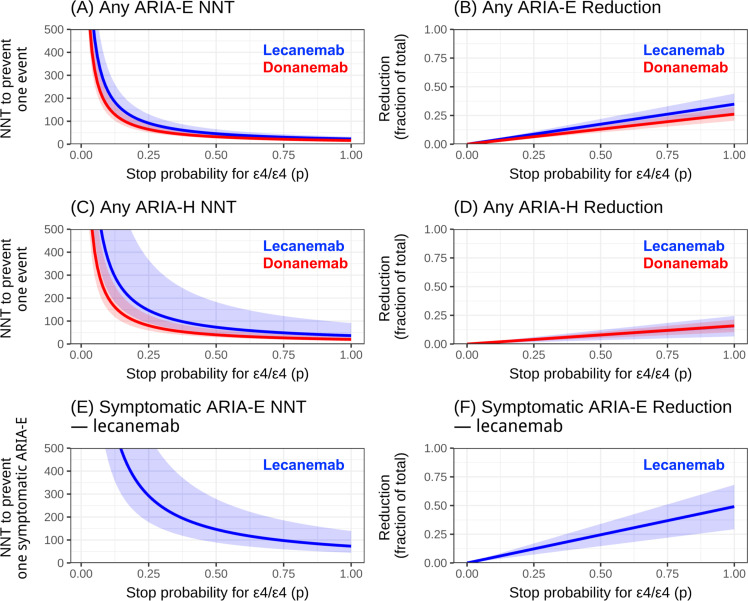
Relationship between ε4/ε4 Stop Probability (p) and ARIA Prevention Metrics. The figure shows the calculated Number Needed to Test (NNT) to prevent one ARIA event (left column; A, C, E) and the corresponding fractional reduction of total ARIA events (right column; B, D, F), plotted against the probability of withholding treatment for *APOE* ε4-homozygotes (p). (A) NNT and (B) Reduction for Any ARIA-E. (C) NNT and (D) Reduction for Any ARIA-H. (E) NNT and (F) Reduction for Symptomatic ARIA-E (lecanemab only). Lecanemab (blue) and donanemab (red) are compared. Solid lines represent median estimates, and shaded areas represent the 95 % Credible Intervals. Abbreviations: NNT, number needed to test; ARIA, Amyloid-Related Imaging Abnormalities; ARIA-E, ARIA-Edema/effusion; ARIA-H, ARIA-Hemorrhage/microhemorrhage/siderosis.

Fig. 1A illustrates how the NNT to avoid one case of any kinds of ARIA-E changes for lecanemab and donanemab, depending on the stopping policy parameter p. At *p* = 1, the median NNT to avert one any ARIA-E event was ∼20–30 for lecanemab and ∼15–25 for donanemab. At *p* = 0.50, NNTs increased to ∼50+ and ∼30–40 respectively; at *p* = 0.25, to ∼90–100 and ∼70–80. CrIs for the two drugs overlapped. Reduction fraction was approximately 30–40 % (Fig. 1B). These figures imply that even under strict withholding (*p* = 1), total ARIA-E can only be reduced by roughly 10–30 %, because events still occur in non-ε4/ε4 patients. This illustrates that *APOE*-based withholding is inherently a blunt instrument at the population level: because the non-ε4/ε4 group is much larger, a substantial share of total ARIA (and MRI monitoring burden) will persist even under the most aggressive ε4/ε4 stopping policy. For any kinds of ARIA-H (Fig. 1C,D), NNT at *p* = 1 was ∼37 (lecanemab) and ∼20 (donanemab); at *p* = 0.25, ∼150 and ∼80. Drug differences were not definitive (CrI overlap).

### 3.2 NNT for avoiding symptomatic ARIA-E (lecanemab)

Fig. 1E,F illustrate how the NNT to avoid one case of symptomatic ARIA-E changes, only for lecanemab, depending on the stopping policy parameter p. For symptomatic ARIA-E, the NNT remained large: >250 at *p* = 0.25, ∼150 at *p* = 0.50, and ∼70–90 at *p* = 1; uncertainty was wide. Even under *p* = 1, symptomatic ARIA-E could not be eliminated because events in non-ε4/ε4 persist.

## 4 Discussion

Our analysis quantified the NNT to prevent one ARIA event, calculated as a function of p-a parameter representing the probability that anti-amyloid therapy is withheld following the identification of *APOE* ε4/ε4 genotype. This parameter p is not a theoretical policy choice; it reflects real-world practice at a given site. Under the most conservative scenario (a universal discontinuation policy, *p* = 1), the NNT to prevent one any-ARIA event was approximately 20–40. While this figure appears efficient, its impact on the total population burden is modest. In that sense, *APOE* testing functions as a modest lever for direct safety improvement: it can reduce a portion of ARIA burden, but it cannot substitute for robust MRI monitoring infrastructure and careful clinical selection, which remain essential regardless of genotype. This policy would reduce the total number of ARIA events by only 10–30 %, as ARIA occurring in non-homozygotes are, by definition, not prevented by this genotype-specific strategy.

This modest population-level benefit must be interpreted in the context of ARIA's typical clinical profile. The majority of ARIA events are asymptomatic, and symptomatic cases are often mild and transient [2,3]. Based on this endpoint (any ARIA), the safety benefit of *APOE* testing appears limited, and a policy of universal treatment discontinuation for ε4-homozygotes is difficult to justify. This conclusion holds even when reframing the endpoint as the prevention of additional, unscheduled MRI scans. If we assume one ARIA event leads to an average of 1–2 additional MRIs, the NNT(MRI) would be 10–20. In a clinical setting, this small reduction may not significantly alter overall resource capacity, which is already burdened by numerous scheduled monitoring scans.

The interpretation changes when focusing on symptomatic ARIA, the primary outcome clinicians and patients wish to avoid. For symptomatic ARIA-E (using lecanemab data), the NNT remained high, at approximately 80 (at *p* = 1), though this policy could prevent up to 50 % of such events. The NNT of 80 seems inefficient, but its operational implications are more favorable. If a single symptomatic event requires an average of four urgent MRIs, the NNT_MRI_ becomes 20 (80/4). This is a meaningful benefit, as urgent MRIs are disproportionately burdensome on institutional resources and staff. This highlights that the testing benefit may be primarily operational (i.e., managing resource capacity) rather than a fundamental reduction in absolute clinical risk.

A crucial point is that p is not an *a priori* variable set by guidelines, but a *posterior* parameter that emerges from the cumulative clinical practice at a specific site. It reflects the local risk tolerance of clinicians and patients, communication practices, and shared decision-making outcomes. It is not feasible to assume a "neutral" or "standard" practice (e.g., *p* = 0.5) exists without robust, real-world registry data. Consequently, any calculated NNT is only meaningful when interpreted relative to the specific, realized p from which it was derived.

This relationship clarifies the appropriate use of these metrics. Clinical practice (policy) is the independent variable; p and NNT are dependent outcomes. If a site's clinical practice changes (e.g., due to new evidence or different risk counseling), p will change, and NNT will follow. In contrast, attempting to alter clinical practice (i.e., *p*) merely to achieve a desired NNT or statistical threshold would reverse the usual clinical decision logic and is not appropriate. When viewed strictly as a safety tool to prevent adverse events, the impact of *APOE* testing is modest.

In summary, if the primary goal of *APOE* testing is to withhold treatment from ε4-homozygotes specifically to prevent ARIA, the institution-level utility of the test is currently limited. This is due to two factors: (a) ARIA risk is not exclusively concentrated in homozygotes, limiting the maximum achievable risk reduction, and (b) in current practice, there are few alternative management pathways besides withholding treatment, making it difficult to achieve a high p (i.e., clinicians may be reluctant to withhold treatment entirely). The utility of testing could increase, however, if risk-stratified management protocols become standard-for example, enhanced MRI monitoring for ε4-homozygotes and potentially streamlined monitoring for non-carriers. Such differential pathways would provide clear alternatives, likely increasing p and the test's practical significance.

These operational and statistical considerations are distinct from the test's ethical and legal importance. *APOE* testing is recommended in clinical guidelines to inform risk. Regardless of the NNT, it is a crucial component of shared decision-making (SDM) [12], allowing patients to receive a clear explanation of their genotype-specific risks. In regions where testing is optional, a decision not to test, following appropriate counseling, can be as medically reasonable as a decision to test. The contribution of this analysis is to provide a quantitative framework for *informing* that SDM process, allowing institutions and clinicians to understand the precise safety benefits their specific clinical practice (p) is achieving.

This distinction between informational utility for SDM and direct safety intervention is critical. The *APOE*-ARIA relationship, when viewed from a purely medical safety standpoint, substantially differs from other established pharmacogenomic (PGx) precedents. For example, PGx tests such as *NUDT15* screening before azathioprine initiation (to mitigate high-probability risks like leukopenia [>50 % risk] or severe alopecia [∼90 % risk]) [13] or HLA-B*57:01 testing for abacavir (to prevent severe hypersensitivity reactions) [14] are clinically established because they prevent adverse events that are both severe and highly frequent in carriers. In contrast, the *APOE*-ARIA link involves an adverse event where the majority of cases are asymptomatic or mild and transient. Therefore, *APOE* testing as a standalone safety lever cannot be expected to yield the same definitive reduction in severe events as these other PGx applications.

Our analysis of donanemab utilized data from the pivotal TRAILBLAZER-ALZ2 trial [3], which formed the basis for its initial approval. We acknowledge that since mid-2025, a titration dosing regimen [15], which is associated with a lower ARIA risk, has been becoming more common. This newer dosing data could not be analyzed in our framework, as the corresponding study lacked a placebo comparator arm, which is necessary to calculate the drug-attribuTable risk difference. We can, however, infer the likely impact. Assuming the titration dose primarily lowers the ARIA rates in the active treatment groups (r_hA_ and r_nA_), the resulting RD_h_ would decrease. Because RD_h_ is directly proportional to ARR, the ARR would also decrease, subsequently leading to a *larger* (less favorable) NNT. Therefore, the use of data from the lower-risk titration regimen would likely reinforce, rather than alter, our study’s above discussions.

This study has several limitations. First, our analysis relied on aggregate data from published clinical trial tables. Consequently, we could not model the influence of patient-level confounders or differences in imaging schedules. Second, genotype-stratified data for symptomatic ARIA were not available for all endpoints (e.g., symptomatic ARIA-H), which limits direct, cross-agent comparisons for this critical outcome. Third, the parameter k (average additional MRIs per ARIA event) was an assumption, as robust empirical data for this are lacking. Therefore, the NNT-MRI calculations should be interpreted as illustrative rather than definitive. Moreover, as emphasized in the discussion, p is a post-hoc, institution-specific parameter. The p value realized at one center cannot be generalized to another without caution and transparency regarding local practices. Finally, *APOE* testing and ARIA mitigation are shaped by the local context, such as regulatory rules, payer coverage, and health-system capacity. We do not resolve these policy trade-offs here; instead, we provide quantitative estimates that can support discussion among these stakeholders.

In conclusion, *APOE* testing contributes to ARIA mitigation, but its stand-alone safety impact is limited. Even under a universal discontinuation policy (*p* = 1), the NNT for any ARIA-E was 20–30 (lecanemab) and 15–25 (donanemab), yet this translates to only a 10–30 % reduction in total ARIA events. Symptomatic ARIA-E, the more critical endpoint, remains difficult to prevent (NNT approximately 70–90 at *p* = 1). These findings support positioning *APOE* testing not as a singular, powerful safety lever, but as a practical decision-support tool. Its primary value lies in informing SDM and aiding in operational planning (e.g., managing MRI capacity for symptomatic events). A pragmatic path forward to enhance the test's value involves developing genotype-tailored management protocols-such as stratified MRI monitoring-rather than relying solely on treatment discontinuation. Furthermore, site-level tracking and reporting of their realized p and local ε4-homozygote prevalence (π) would provide the necessary context to translate this analysis into meaningful clinical practice. Because we focus only on risk and operational burden (not clinical benefit), our results should be read as showing that genotype-based withholding has limited direct safety impact, and that *APOE* testing is mainly useful for shared decision-making and operational planning.

## Consent statement

N/A.

## Declaration of the use of generative AI and AI-assisted technologies in scientific writing and in figures, images and artwork

The authors used cloud large language models (ChatGPT and Gemini) for English proofreading. After using these tools, the authors reviewed and edited the content as needed and take full responsibility for the content of the published article.

## Data availability

The data used in this analysis were extracted from published trial reports and are publicly available.

## Funding

This study was supported by AMED Grant Number 24dk0207068 (TI) and 25dk0207075 (KS) and JSPS KAKENHI Grant Number JP24K10653 (RI) and JP25K19014 (KS).

## CRediT authorship contribution statement

**Kenichiro Sato:** Writing – original draft, Visualization, Methodology, Investigation, Formal analysis, Conceptualization. **Yoshiki Niimi:** Writing – review & editing, Conceptualization. **Masanori Kurihara:** Writing – review & editing, Conceptualization. **Ryoko Ihara:** Writing – review & editing. **Atsushi Iwata:** Writing – review & editing. **Takeshi Iwatsubo:** Writing – review & editing, Supervision.

## Declaration of competing interest

The authors declare the following financial interests/personal relationships which may be considered as potential competing interests: Kenichiro Sato reports financial support was provided by Japan Society for the Promotion of Science. Kenichiro Sato reports financial support was provided by Japan Agency for Medical Research and Development. Ryoko Ihara reports financial support was provided by Japan Society for the Promotion of Science. Takeshi Iwatsubo reports financial support was provided by Japan Agency for Medical Research and Development. The authors’ affiliation, “Dementia Inclusion and Therapeutics,” is an endorsed course funded by Effissimo Capital Management Pte Ltd. If there are other authors, they declare that they have no known competing financial interests or personal relationships that could have appeared to influence the work reported in this paper.

## References

[1] Ramanan V.K., Armstrong M.J., Choudhury P., Coerver K.A., Hamilton R.H., Klein B.C., Wolk D.A., Wessels S.R. (2023). Jones LK Jr; AAN Quality Committee. Antiamyloid monoclonal antibody therapy for Alzheimer Disease: emerging issues in neurology. Neurology.

[2] van Dyck C.H., Swanson C.J., Aisen P., Bateman R.J., Chen C., Gee M., Kanekiyo M., Li D., Reyderman L., Cohen S., Froelich L., Katayama S., Sabbagh M., Vellas B., Watson D., Dhadda S., Irizarry M., Kramer L.D., Iwatsubo T. (2023). Lecanemab in early Alzheimer's Disease. N Engl J Med.

[3] Sims J.R., Zimmer J.A., Evans C.D., Lu M., Ardayfio P., Sparks J., Wessels A.M., Shcherbinin S., Wang H., ES Monkul Nery, Collins E.C., Solomon P., Salloway S., Apostolova L.G., Hansson O., Ritchie C., Brooks D.A., Mintun M., DM; Skovronsky (2023). TRAILBLAZER-ALZ 2 investigators. Donanemab in early symptomatic Alzheimer disease: the TRAILBLAZER-ALZ 2 randomized clinical trial. JAMA..

[4] Dunn B., Stein P., Cavazzoni P. (2021). Approval of Aducanumab for Alzheimer Disease-the FDA's perspective. JAMA Intern Med.

[5] Zimmer J.A., Ardayfio P., Wang H., Khanna R., Evans C.D., Lu M., Sparks J., Andersen S., Lauzon S., Nery E.S.M., Battioui C., Engle S.E., Biffi A., Svaldi D., Salloway S., Greenberg S.M., Sperling R.A., Mintun M., Brooks D.A., Sims J.R. (2025). Amyloid-related imaging abnormalities with Donanemab in early symptomatic Alzheimer disease: secondary analysis of the TRAILBLAZER-ALZ and ALZ 2 randomized clinical trials. JAMA Neurol.

[6] Cummings J., Apostolova L., Rabinovici G.D., Atri A., Aisen P., Greenberg S., Hendrix S., Selkoe D., Weiner M., Petersen R.C., Lecanemab S.S. (2023). Appropriate use recommendations. J Prev Alzheimers Dis.

[7] Rabinovici G.D., Selkoe D.J., Schindler S.E., Aisen P., Apostolova L.G., Atri A., Greenberg S.M., Hendrix S.B., Petersen R.C., Weiner M., Salloway S., Cummings J. (2025). Donanemab: appropriate use recommendations. J Prev Alzheimers Dis.

[8] Sato K., Niimi Y., Ihara R., Iwata A., Nemoto K., Arai T., Higashi S., Igarashi A., Kasuga K., Akiyama H., Awata S., Ikeda M., Iwatsubo T. (2025). Real-world lecanemab adoption in Japan 1 year after launch: insights from 311 specialists on infrastructure and reimbursement barriers. Alzheimers Dement.

[9] EMA (https://www.ema.europa.eu/en/medicines/human/EPAR/leqembi): Accessed on November 10, 2025.

[10] MHRA (https://products.mhra.gov.uk/product/?product=LEQEMBI): Accessed on November 10, 2025.

[11] McAlister F.A. (2008). The "number needed to treat" turns 20–and continues to be used and misused. CMAJ.

[12] Elwyn G., Frosch D., Thomson R., Joseph-Williams N., Lloyd A., Kinnersley P., Cording E., Tomson D., Dodd C., Rollnick S., Edwards A., Barry M. (2012). Shared decision making: a model for clinical practice. J Gen Intern Med.

[13] Relling M.V., Schwab M., Whirl-Carrillo M., Suarez-Kurtz G., Pui C.H., Stein C.M., Moyer A.M., Evans W.E., Klein T.E., Antillon-Klussmann F.G., Caudle K.E., Kato M., Yeoh A.E.J., Schmiegelow K., Yang J.J. (2019). Clinical Pharmacogenetics Implementation Consortium Guideline for thiopurine dosing based on TPMT and NUDT15 genotypes: 2018 update. Clin Pharmacol Ther.

[14] Martin M.A., Hoffman J.M., Freimuth R.R., Klein T.E., Dong B.J., Pirmohamed M., Hicks J.K., Wilkinson M.R., Haas D.W., Kroetz D.L. (2014). Clinical Pharmacogenetics Implementation Consortium. Clinical Pharmacogenetics Implementation Consortium Guidelines for HLA-B genotype and Abacavir dosing: 2014 update. Clin Pharmacol Ther.

[15] Wang H., Serap Monkul Nery E., Ardayfio P., Khanna R., Otero Svaldi D., Gueorguieva I., Shcherbinin S., Andersen S.W., Hauck P.M., Engle S.E., Brooks D.A., Collins E.C., Fox N.C., Greenberg S.M., Salloway S., Mintun M.A., Sims J.R (2025). Modified titration of donanemab reduces ARIA risk and maintains amyloid reduction. Alzheimers Dement.

